# Global Scientific Research on SARS-CoV-2 Vaccines: A Bibliometric Analysis

**DOI:** 10.22074/cellj.2021.7794

**Published:** 2021-10-30

**Authors:** Fakher Rahim, Aida Khakimova, Ammar Ebrahimi, Oleg Zolotarev, Fatemeh Rafiei Nasab

**Affiliations:** 1. Thalassemia and Hemoglobinopathy Research Centre, Ahvaz Jundishapur University of Medical Sciences, Ahvaz, Iran; 2. Department of Development of Scientific and Innovation Activities, Russian New University, Moscow, Russia; 3. Department of Medical Biotechnology, School of Paramedicine, Guilan University of Medical Sciences, Rasht, Iran; 4. Department of Information Systems in Economics and Management, Russian New University, Moscow, Russia; 5. Department of Scientometrics, Deputy of Research and Technology Affairs, Ahvaz Jundishapur University of Medical Sciences, Ahvaz, Iran

**Keywords:** Bibliometric Analysis, COVID-19/SARS-CoV-2, Vaccine

## Abstract

**Objective:**

We performed this bibliometric analysis to identify global scientific research on the SARS-CoV-2 vaccines.

**Materials and Methods:**

This bibliometric analysis study inclusive search of English-language publications related to the SARS-CoV-2 vaccines was conducted in the Scopus, PubMed, and Dimensions databases without year limitations. The results of bibliometric analysis comprised a time-dependent citation density trend, the name of the journal, journal impact factor (IF), year of publication, type of article, category, subscription or affiliation, co-authorship, and co- occurrence network.

**Results:**

A study of the scientific literature from three databases (Scopus, PubMed, Dimensions) shows that investigators have focused more on studying the structure of the coronavirus at different levels (organismic, cellular, and molecular). In addition, the method of virus penetration into the cell and features of the influence of coronavirus on animals are well-studied. Various methods and strategies are being used to develop the vaccines, including both animal-tested methods and computer models. The Dimensions database is the most representative in terms of coverage of research on development of the SARS-CoV-2 vaccines.

**Conclusion:**

This research is a scientific investigation based on bibliometric analysis of papers related to the SARS-CoV-2 vaccines. The Dimensions database provides the most representative research coverage on the creation of a vaccine against coronavirus. It is characterized by a large number of formed verbose terms (length of more than four words) related to coronavirus, which makes it possible to track trends in the development of methods for creating a vaccine.

## Introduction

The COVID-19 outbreak has caused many economic and psychological effects, and many casualties (1). This virus has spread worldwide with indescribable speed over a short period of time. According to experts, the COVID-19 pandemic could last for years; hence, numerous scientists worldwide are working to eradicate this virus as soon as possible (2). After the increase in cases and global spread, the World Health Organization (WHO) announced that the new coronavirus is the sixth public health emergency worldwide (3).

Diagnosis of a COVID-19 infection is generally based on laboratory and radiological assessments, and radiological examinations are extremely important in early diagnosis and treatment of this disease (4). Severe lung damage due to COVID-19 infection has resulted in high mortality rates in patients who are infected and requirements for mechanical ventilation are also high (5). There is no specific antiviral treatment for the COVID-19 infection, and the mainstay is supportive care that includes sustaining vital signs, oxygen therapy, and the reduction of complications such as multiple organ dysfunction and failure (6). Due to the lack of standard treatment and effective vaccines for this infection, prevention of infection is the best recommendation. 

A vaccine is a biological preparation that protects the body against certain infectious diseases. Vaccines usually contain a pathogen, which is similar to the microorganism that causes the disease and is often obtained from a sample of weak or dead microbes, toxins, or one of its surface proteins. Vaccines are either for prevention (to prevent or help cure an infection by a natural or artificial pathogen) or for treatment (such as a cancer vaccine that has not yet been discovered). SARS-CoV-2 vaccines fall into two groups of genetic vaccines that use one or more of the genes of the coronavirus to stimulate an immune response or a vaccine that carries the virus where the virus is used to deliver the corona virus gene to cells and stimulate an immune response (7). Studies for SARS-CoV-2 vaccines development are ongoing; despite significant progress in vaccine development, challenges still exist (8). The development of a safe, effective vaccine is a long and complicated process that typically takes 10 to 15 years (9). Currently more than 100 candidates for the SARS-CoV-2 vaccines are in various stages of development and a small number are in the early phases of human clinical trials (10). SARS-CoV-2 vaccines approved by the WHO are in clinical trials (11) and a close competition exists between them to achieve a positive result. 

In October, 2020, the US Food and Drug Administration (FDA) approved an antiviral drug, Remdesivir (GS-5734), for the treatment of patients hospitalised with COVID-19. This is the first and only approved drug for treatment of COVID-19 in the United States. Remdesivir, an intravenous (IV) injectable drug, inhibits the substances that increase viral replication. Experts warn against the simultaneous use of this drug with hydroxychloroquine because hydroxychloroquine inhibits the therapeutic effects of Remdesivir (12). Remdesivir was originally developed to treat Ebola, but it was not effective and eventually discarded. This appears to be happening again in patients with COVID-19 infection. 

The results of recent studies where Remdesivir was used to reduce the complications of COVID-19 infection showed that this drug had little effect on patient recovery (13).

Bibliometric analysis is a tool to determine the status of research conducted in a particular field (14). Trends and possible gaps in knowledge play an important role in management and decision making in science and technology (15). Bibliometric analysis mainly allows the development of analytical methods and bibliometric indicators from statistical criteria, and it is a tool that manages information records related to publications, citations, patents, reports, etc. (16). This analysis also provides additional information about data such as author(s), affiliation(s), and keywords, in addition to integrating information to develop research areas on a specific topic or disciplines.

Despite rapid response from scientists during COVID-19 pandemic, vaccines and antibody protection are still out of reach; however, in acute cases, the US FDA may allow emergency use of promising vaccines that have not yet fully passed safety tests (17). However, for at least six months, researchers will not know the benefits of a vaccine. People exposed to the virus should hope to strengthen their immune system and receive supportive care from doctors and nurses to fight this disease.

The necessity and importance of the present research is that the findings, which include articles from a time period on SARS-CoV-2 vaccines, can show the status of research in this field, reference resources, and reveal the strengths and weaknesses of these researches. Future researchers can fill the information gap in this field by conducting research. In this study, we intend to identify global scientific research on SARS-CoV-2 vaccines by using bibliometric analysis. 

## Materials and Methods

### Search method and strategy

We performed a search in the Scopus database by October 2020 based on a protocol published by Mecenas et al. (18) in 2020. We searched the Scopus database for titles, abstracts, and keywords. A total of 1659 publications were found for 2019-2020 (1657 publications for 2020, 1 publication for 2019). We also performed a search in the PubMed database on 22/07/2020 and located 6727 articles, of which 6225 were published from 01/01/2019- 31/07/2020. We searched the Dimensions database for "Vaccine coronavirus" in titles and abstracts and found 2326 publications for 2019-2020 (2169 publications for 2020, 157 publications for 2019). Of these, publications from PubMed - 1289. Table S1 (See Supplementary Online Information at www.celljournal.org) provides the detailed search strategies for each selected database.

### Data extraction

Data collection in this study was conducted with a number of articles and by using a researcher-made form appropriate to the objectives of the research. The studied variables included: number of universities, number of journals in each university, number of articles published, number of citations, countries, publication types, first author and contact author, and the number of articles published in each of the fields of SARS-CoV-2 vaccines. We used the VOSviewer toolkit to conduct a co-occurrence analysis for the Scopus, PubMed, and Dimensions databases. An assessment was made of the intensity of the use of one term with another. The minimum threshold for cluster formation was set in a different number of terms for different databases.

### Statistical analysis

For data processing, Excel software and descriptive statistics indicators such as mean value were used. (version 1.6.15, Leiden, The Netherlands) was used for visualization. A P<0.05 was considered as significant. 

## Results

### Citation analysis

Table 1 lists the ten top-cited results. There were 1897 citations and three papers had at least 200 citations. The first paper had 514 citations and was published by Wrapp et al. (19) in the Proceedings of Department of Molecular Biosciences, University of Texas at Austin (Austin, TX, USA). 

**Table 1:** The top-cited studies on SARS-CoV-2 vaccines


	Title	Citation type	PMID	DOI	IF

1	Cryo-EM Structure of the 2019-nCoV Spike in the Perfusion Conformation	514Original	32075877	10.1126/science.abb2507	41.845
2	Structure, Function, and Antigenicity of the SARS-CoV-2 Spike Glycoprotein	390Original	32155444	10.1016/j.cell.2020.02.058	38.637
3	Angiotensin-converting Enzyme 2 (ACE2) as a SARS-CoV-2 Receptor: Molecular Mechanisms and Potential Therapeutic TargetOpen Access	232Review	32125455	10.1007/s00134-020-05985-9	17.679
4	Drug Treatment Options for the 2019-New Coronavirus (2019-nCoV)Open Access	178Communication	31996494	10.5582/bst.2020.01020	1.690
5	Characterization of Spike Glycoprotein of SARS-CoV-2 on Virus Entry and Its Immune Cross-reactivity with SARS-CoV	138Original	32221306	10.1038/s41467-020-15562-9	12.121
6	Preliminary Identification of Potential Vaccine Targets for the COVID-19 Coronavirus (SARS-CoV-2) based on SARS-CoV Immunological StudiesOpen Access	114Original	32106567	10.3390/v12030254	3.816
7	Structure of Mpro from SARS-CoV-2 and Discovery of Its InhibitorsOpen Access	96Original	32272481	10.1038/s41586-020-2223-y	42.778
8	Characterization of the Receptor-binding Domain (RBD) of 2019 Novel Coronavirus: Implication for Development of RBD Protein as a Viral Attachment Inhibitor and Vaccine	80Original	32203189	10.1038/s41423-020-0400-4	8.484
9	A SARS-CoV-2 Protein Interaction Map Reveals Targets for Drug RepurposingOpen Access	78Original	32353859	10.1038/s41586-020-2286-9	42.778
10	Emergence of Genomic Diversity and Recurrent Mutations in SARS-CoV-2	77Review	32387564	10.1016/j.meegid.2020.104351	2.611


PMID; PubMed ID, DOI; Digital object identifier, and IF; Impact factor 2019.

### Journals

The "Journal of Bimolecular Structure and Dynamics"has an extremely large number of contributions to COVID-19 research with 33 publications followed by "Nature" with 15 papers and "Medical Hypotheses" with 14 papers. Altogether, the ten highest-ranking journals issued 129 articles, which accounted for 17.25% of all publications in this area from a total of 748 (100%) publications. Table 2 lists the top ten funding agencies and highest-ranking journals. 

A total of 30 (4.02%) publications were supported by the National Natural Science Foundation of China and 29 were funded by the National Institutes of Health (03.88%) (Table 2).

### Journal impact factor

Impact factors (IFs) for the journals with the top-cited articles ranged from 1.322 to 42.778 (median: 3.324). Overall, 52 of the top-cited studies were published in journals that had IFs above 15 (Table 2). Finally, the correlations between the number of top-cited papers and journal IFs did not show any statistical significance (P>0.05). 

### Publication type

Overall, 1868 articles were cited 12 675 times and 1089 review papers were cited 9710 times. The articles were had a higher average citation per study (429 times) compared to the review papers, which were cited 378 times. Medicine was the most popular research category, followed by biochemistry, genetics, immunology, and microbiology. In terms of research category, there were 60 published studies that pertained to clinical research, of which 11 papers were about therapeutic vaccines (eight full papers and three protocols, including nine that pertained to phase I/II research studies and two phase III studies) (20-30). Table S2 (See Supplementary Online Information at www.celljournal.org) provides detailed information about these clinical trials.

### Language and year

All of these papers were published in English from 2019 to 2020.

**Table 2:** Top funding sources and journals for studies about SARS-CoV-2


No.	Field		IF (2019)	N	%
			Journal		

1	Journal of Biomolecular Structure and Dynamics		3.22	33	4.41
2	Nature		42.778	15	2.01
3	Medical Hypotheses		1.322	14	1.87
4	Frontiers in Immunology		6.429	11	1.47
5	Journal of Medical Virology		2.049	11	1.47
6	Cell		38.637	10	1.34
7	Chaos Solitons and Fractals		3.380	10	1.34
8	Vaccine		3.269	9	1.2
9	Cell Host and Microbe		15.923	8	1.07
10	Diabetes and Metabolic Syndrome Clinical Research and Reviews		1.940	8	1.07
Funding sponsors					
1	National Natural Science Foundation of China		-	30	4.02
2	National Institutes of Health		-	29	3.88
3	National Institute of Allergy and Infectious Diseases		-	16	2.14
4	Bill and Melinda Gates Foundation		-	10	1.34
5	National Basic Research Program of China (973 Program)		-	8	1.07
6	National Science Foundation		-	7	0.94
7	Chinese Academy of Sciences		-	6	0.8
8	National Research Foundation of Korea		-	6	0.8
9	Science and Engineering Research Board		-	6	0.8
10	Welcome Trust		-	6	0.8


N; Number, %; Percentage, and IF; Impact factor.

### Country

The United States produced the most publications with 190 papers (25.40%), followed by India (126 publications, 16.84%) and China (88 papers, 11.76%). The United States ranked first in terms of gross domestic product (GDP) and articles per million population, with 0.009 articles per billion GDP (Table 3).

### Co-authorship network by authors

In the Scopus database, we found 1659 articles of which there were 6545 authors. From the 6545 authors, 59 authors had at least five published papers.

The co-authorship Scopus network included 59 authors in nine clusters. However, clusters 8 and 9 included only one author (Fig .S1A, See Supplementary Online Information at www.celljournal.org). Table S3 (See Supplementary Online Information at www.celljournal.org) lists clusters 1-7. We located 6225 articles in PubMed and 26 509 authors in the corpus. For comparison with the co-authorship network obtained for the corpus from Scopus, we selected 59 authors who had at least six publications. The co-authorship PubMed network included 59 authors in 19 clusters (Fig .S1B, See Supplementary Online Information at www.celljournal.org). There were 2326 articles from the Dimensions database with 10 356 authors in the corpus. We selected 59 of the most cited authors who had at least five publications to compare the co-authorship network obtained for the corpora from Scopus and PubMed. All authors of the articles were selected for consideration. The co-authorship Dimensions-network included 59 authors in ten clusters (Fig .S1C, See Supplementary Online Information at www.celljournal.org).

**Fig.1:**
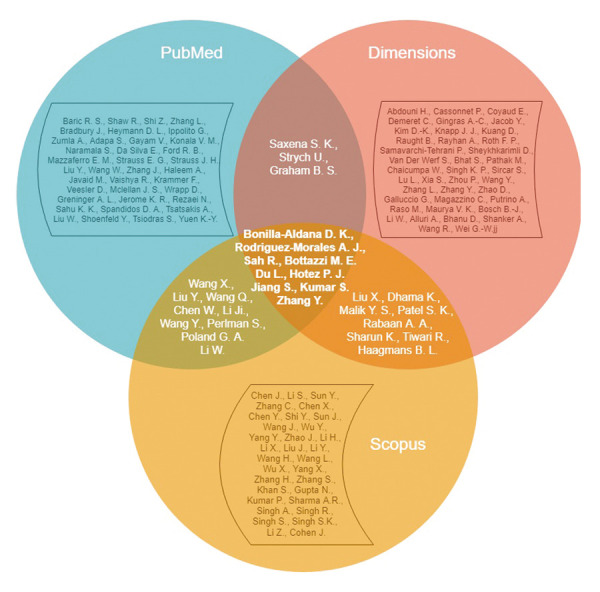
Comparison of the most cited authors of articles in three databases (Scopus, PubMed, Dimensions) that pertain to the vaccine against SARS-CoV-2.

Only nine authors were present in the three text corpora (Bonilla-Aldana D.K., Rodriguez-Morales A.J., Sah R., Bottazzi M.E., Du L., Hotez P.J., Jiang S., Kumar S., and Zhang Y.) (Fig .1). 

**Table 3:** Adjusted gross domestic product (GDP) and articles that pertained to studies on the SARS-CoV-2 vaccines per million population


No.	Country/territory	Number	(%)	N per million population	N per billion GDP

1	United States	190	25.4	5.8	0.009
2	India	126	16.84	9.31	0.04
3	China	88	11.76	6.31	0.006
4	United Kingdom	59	7.89	8.87	0.02
5	Italy	53	7.09	8.77	0.02
6	Australia	27	3.61	1.08	0.01
7	Germany	26	3.48	3.15	0.006
8	Pakistan	26	3.48	1.22	0.08
9	Canada	25	3.34	6.74	0.01
10	France	23	3.07	3.73	0.009


### Co-authorship network by organizations

Out of 5185 organizations of the corpus in the Scopus database, five had connections and published at least five scientific papers. There were 34 organizations that had at least three publications. We identified 34 organizations that formed 22 clusters in the co-authorship network (Fig. S2A, See Supplementary Online Information at www. celljournal.org).

Out of 15 718 organizations of the corpus from PubMed, 82 had at least three publications. We selected 34 organizations to compare with the co-authorship network obtained for the corpus from Scopus. The co-authorship PubMed network included 34 organizations in 14 clusters (Fig .S2B, See Supplementary Online Information at www.celljournal.org). Of the 1684 organizations of the corpus from the Dimensions database, 34 organizations had at least 12 publications. The 34 organizations formed a co-authorship network of six clusters (Fig .S2C, See Supplementary Online Information at www.celljournal. org). Table S4 (See Supplementary Online Information at www.celljournal.org) shows the first six clusters. We compared the composition of clusters of co-authorship networks by organizations obtained by the corpora from the Scopus, PubMed and Dimensions databases in Table S4 (See Supplementary Online Information at www. celljournal.org). 

### Co-occurrence network map of keywords 

A co-occurrence analysis of keywords was performed that displayed the existing links between keywords used in the publications. In the central part of the map, the terms most frequently encountered in publications are displayed. The keywords/terms were extracted from the title field. A term is presented as a chain of elements (nouns with definitions) with a noun at the end of the phrase [van Eck and Waltman, (31)] (Fig. S3, See Supplementary Online Information at www. celljournal.org). 

**Fig.2:**
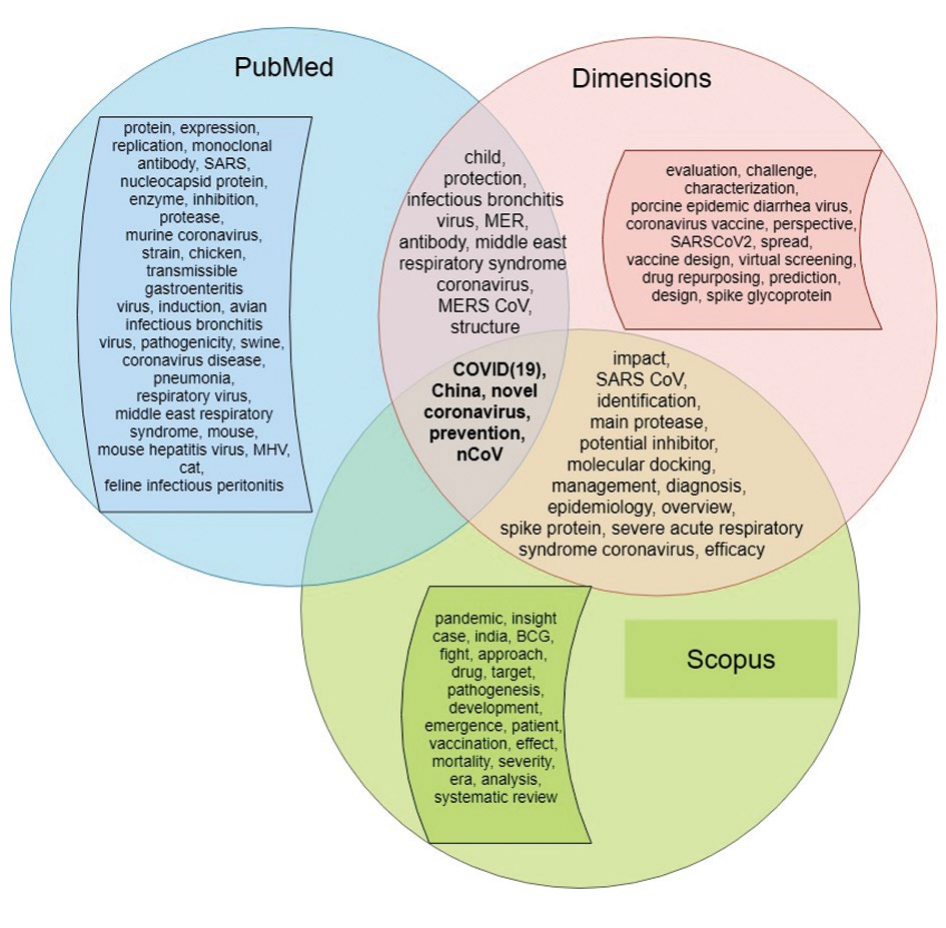
A comparison of the key terms from different corpora (Scopus, PubMed, Dimensions).

In our analysis of the corpus from Scopus, we set the threshold of the minimum number of keyword occurrences at ten. This analysis resulted in 39 keywords out of a total of 3625 (Table S5, See Supplementary Online Information at www.celljournal.org). For correct comparison, we choose the same number of terms in the corpus of the Dimensions database. A threshold for the minimum number of keyword occurrences was set at 15. The analysis resulted in 71 keywords out of a total of 4865. We used VOSviewer, which automatically extracted 40% of the least relevant terms; therefore, we chose 43 terms. The common terms “use”, “India”, “time”, and “knowledge” were excluded from the general list (Table S6, See Supplementary Online Information at www. celljournal.org). For correct comparison, we chose the same number of terms in the corpus of the PubMed database. We set the threshold of a minimum number of keyword occurrences at 90. The analysis resulted in 88 keywords out of a total of 26 884. VOSviewer filtered out about 40% of the terms; hence, 53 terms were chosen. We excluded 14 common terms such as “Saudi Arabia”, “vitro”, “South Korea”, and “lesson” from the general list (Table S7, See Supplementary Online Information at www.celljournal.org). Only five terms were present in the three text corpora (COVID-19), China, novel coronavirus, prevention, nCoV) (Fig .2).

The previous experiment was limited in the number of terms. In addition, manual filtering of terms might have affected the result. So, we repeated the experiment with more terms. We choose the conditions of mapping (minimal number of occurrences, minimal cluster size) such that the number of terms approximated 450 and the number of clusters was four (Fig .S4, See Supplementary Online Information at www.celljournal.org).

For correct comparison, we choose the same number of terms in the corpora of the PubMed and Dimensions databases. A threshold of a minimum number of keyword occurrences equal to four for PubMed and three for Dimensions was set. The analysis resulted in 783 keywords out of a total of 10 784 for PubMed and 577 out of 4865 for Dimensions, except for the 40% that were deleted by VOSviewer. Finally, we chose 462 terms for PubMed and 459 terms for the Dimensions database. We did not exclude any terms from the final list. In order to have four clusters, we set limits of at least 60 words in a cluster for PubMed and 80 for the Dimensions database.

The main keywords for each of the four clusters (Top-20) from term co-occurrence maps (rank based on total link strength) are presented in Table S8 (See Supplementary Online Information at www.celljournal.org) for the Scopus database, Table S9 (See Supplementary Online Information at www.celljournal.org) for the PubMed database, and Table S10 (See Supplementary Online Information at www.celljournal.org) for the Dimensions database.

**Fig.3:**
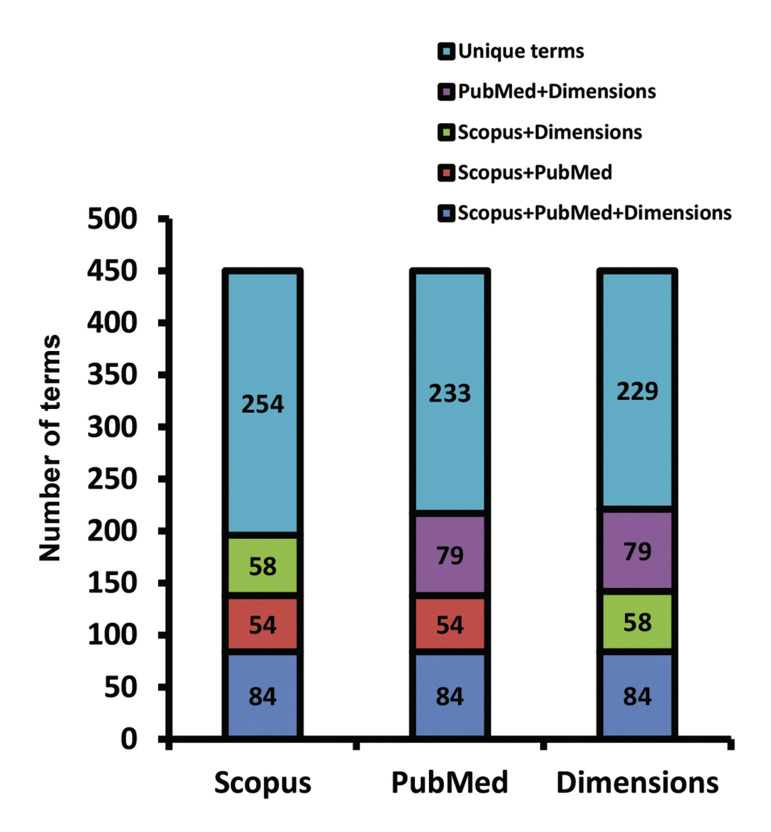
The ratio of repetitive and unique terms in different corpora. There were a total of 450 units for all corpora.

Table S10 (See Supplementary Online Information at www.celljournal.org) presents a comparison of the received terms (450 units) from different corpora. The common vocabulary, general scientific vocabulary, and general medical vocabulary were excluded (Fig .3).

We compared the names of coronavirus diseases used in the resultant text corpora (Table S11, See Supplementary Online Information at www.celljournal.org) and analysed the vocabulary of the terms. We divided the terms into the following groups: i. Common vocabulary, ii. General scientific vocabulary, iii. General medical vocabulary, and iv. Thematic vocabulary. The first group included the following terms: Germany, entry, 21^st^ century, general population, Hubei Province, policy, adult, age, era, future perspective, human, nature, social media, Africa, alternative, communication, community, decision, delay, February, belief, adolescent, adoption, climate change, difference, human right, seasonality, Seattle, disruption, billion compound, chapter, characteristics, characterization, female, goal, Google Scholar, government, guidance, mankind, market, performance, period, week, whole world, Wuhan city, emergence, environment, worldwide, and Wuhan.

The second group included the following terms: discovery, comparison, assessment, bibliometric analysis, biomedical research, correlation, systematic review, cross-sectional study, meta-analysis, current knowledge, validation, current review, observational study, current study, database, further investigation, mathematical model, publication, PubMed, comparative analysis, mathematical assessment, mathematical prediction, computational, deep learning, computational approach, science, scientist, computational investigation, and computational method.

The third group included the following terms: diagnostic, clinical manifestation, cancer patient, case report, silico approach, human health, clinical consideration, clinical course, clinical features, clinical implication, clinical management, hospitalisation, hospitalised child, prophylactic, hospitalised patient, blood, patients, breath, morbidity, mortality, therapeutic approach, therapeutic strategy, cancer, clinical experience, clinical sample, cardiovascular disease, clinical data, clinical evidence, clinical presentation, clinical study, healthcare, healthcare system, healthcare worker, supportive care, clinical symptom, clinical treatment, clinical use, and clinician. Terms from the fourth group are presented in Table S12 (See Supplementary Online Information at www. celljournal.org). 

Figure S5 (See Supplementary Online Information at www.celljournal.org) shows the relationship of the terms of each group between corpora from the different databases. The most common terms in publications related to research in the field of vaccine and coronavirus was “sars cov” in the PubMed database, which was organized based on the time of appearance (Fig .S6, See Supplementary Online Information at www.celljournal. org).

## Discussion

This is the first bibliometric research that summarizes numerous characteristics of the investigations of the SARS-CoV-2 vaccines. An understanding of the features of global researches on SARS-CoV-2 vaccines may be beneficial. In this bibliometric study, we reviewed the literature from three databases - Scopus, Dimensions, and PubMed. We identified 1659 articles from Scopus and 6545 authors in the corpus, of which 59 authors had at least five publications. The co-authorship network includes these authors in nine clusters.

For 6225 articles from PubMed, 26 509 authors were listed in the corpus with the co-authorship network that includes 59 authors in 19 clusters. Moreover, for 2326 articles from the Dimensions database, 10 356 authors were in the corpus, of which 59 authors who had at least five publications with the co-authorship network of ten clusters were included. As can be deduced from the results, although there have been many studies on the SARS-CoV-2 vaccine, as well as number of vaccine showed acceptable efficacy against SARS-CoV-2; therefore, we cannot with certainty expect a fully safe and effective vaccine against this disease, especially for various age groups and various viral strains (32).

To our surprise, we found that that at most, there were 9 authors duplicated in all three corpora of the publications. Scopus and PubMed had nine, whereas Scopus and Dimensions had eight, and PubMed and Dimensions had three mutual authors. There were 33 (55.9%) non-recurring authors in the Scopus-network, 37 (62.7%) in the PubMed network, and 39 (66.1%) in the Dimensions network. It should be noted that Chinese and Indian authors prevailed among Scopus authors. There is a prevalence of European authors in PubMed and the Dimensions database is comprised of European, Chinese, and Indian authors. 

To date, there are more than 300 approved candidates for the SARS-CoV-2 vaccines, and 32 have already undergone clinical trials. In addition, the paradox of vaccine production has been raised in some countries (33). Vaccines can help prevent the spread of disease by stimulating the immune system. Our knowledge of COVID-19 is far less than our ignorance, and the complexity of this disease makes us think more deeply about a vaccine. In the first stage, the vaccine is tested on a small number of subjects in order to prove that it is safe. In the second phase, the vaccine will be tested on a larger number of patients to evaluate its effectiveness. Both safety and efficacy of a vaccine are very important and vital (34). 

The organizations that were identified in the mapping of co-authorship also differed for the three corpora. Organizations that met twice were: University College London, Fudan University, University of Washington, Tehran University of Medical Sciences (Tehran, Iran), Ohio State University, and Yale University. No organization met three times. In a preliminary experiment, our comparison showed considerable variation in terminology. Common words comprised disease names (COVID19, novel coronavirus, nCoV), disease prevention, and country of origin (China). Although unlikely, the COVID-19 outbreak could abruptly end before a safe and effective vaccine is available; therefore, we must continue our efforts to find such vaccines in order to be prepared to fight this disease if an outbreak recurs (35). Given that all scientists and research and development centres are in a race and competition to develop the SARS-CoV-2 vaccines, it is necessary to accelerate and streamline that process because a vaccine may be the only approach that enables the development of immunity to SARS-CoV-2 across a population (36). 

As a result, scientific biomedical information databases are often used by physicians and researchers. In this article, we compared different aspects of basic biomedical scientific information databases. PubMed is a very significant resource for physicians and researchers, whereas Scopus covers a wider range of journals and citation analysis capabilities compared to the other databases (37). At the same time, both the PubMed and Dimensions corpora overlapped in eight terms. The Scopus and Dimensions corpora overlapped in 13 terms. Both the Scopus and PubMed corpora did not overlap in any of the terms. In order to clarify terminological mapping, we conducted research on a large number of terms. The results of the previous study could have been influenced by human factor because we excluded a number of uninformative terms at the last stage. In the current study, we considered all terms without exception. The main term common to the Scopus and PubMed databases was 2019-COV. The PubMed and Dimensions databases had eight terms in common (new coronavirus, novel coronavirus covid, novel coronavirus SARS-CoV, porcine deltacoronavirus, porcine epidemic diarrhoea virus, SARS CoV2, SARSCoV2 infection, and severe acute respiratory syndrome). Therefore, PubMed had more research with animal models on COVID-19 diseases compared to the other databases (38). In addition, more accurate names of this disease were used in this corpus, which was not surprising given the medical nature of this database.

We analysed the lexical groups of the terms, of which the thematic vocabulary group is of interest as it contains terms related to the field of vaccine development (39). Surprisingly, the Dimensions database had the most topic-specific words (249 words), followed by Scopus (221 words) and PubMed (193 words). However, there were words that were repeated in the three corpora. Among the general terms, there were terms that provided an idea that to design a vaccine toolkit, animal models are essential, using approaches such as immune-informatics, virtual screening, molecular dynamics simulation, which are popular methods for vaccine development so far (40).

The structure of the coronavirus is being studied. Animal models are mentioned in the PubMed and Dimensions corpora. Hence, in term of Scopus terms, penetration of the virus into the cell and the body’s response to the virus were frequent terms. PubMed has frequent immunological terms, but the largest number of specific terms were identified in the Dimensions database. Thus, the corpus from the Dimensions database provided a more complete picture of the research topics for SARS-CoV-2 vaccine development. This could help health policy makers make decisions about incorporating new researches into vaccine development (41).

We attempted to analyse temporal dynamics using the PubMed corpus as an example by taking into account the medical specifics of this database because these collections were less represented in the Dimensions and Scopus databases. The time slot is wider in the PubMed database. Unlike other databases, PubMed contains many articles about previous strains of coronaviruses. Nevertheless, the interval of publication activity was small. Hence, we decided to consider temporal dynamics in future studies.

There were 10874 terms in the PubMed collection. We chose 147 terms because they were the most relevant and popular. The clustering of these terms resulted in ten clusters. There were eight clusters with the minimum cluster size that equalled one.

## Conclusion

This study is a scientific bibliometric analysis of studies on SARS-CoV-2 vaccines. A comparative analysis of scientific literature was carried out for three bases: Scopus, PubMed, and Dimensions. As a result, we determined that tremendous attention is paid to the study of the coronavirus structure at the organismic, cellular and molecular levels. Penetration of the virus into the cells is well-studied. A variety of methods and strategies are being used to develop a vaccine. The features of the influence and development of coronavirus on animals are well understood. Both animal and computer models are being used to create a vaccine for humans. The most representative from the point of view of the coverage of research on the creation of a vaccine against coronavirus is in the Dimensions database. It is characterized by a large number of formed verbose terms (more than four words) related to coronavirus, which makes it possible to track trends in the development of methods for creating a vaccine.

## Supplementary PDF


