# Supplementary material for: Global Scientific Research on SARS-CoV-2 Vaccines: A Bibliometric Analysis
Source: Cell J. 2021 Oct 30;23(5):523–31. doi: 10.22074/cellj.2021.7794 (PMC8588811; doi:10.22074/cellj.2021.7794)
Supplement: Supplementary file 1 [file Cell-J-23-523-s01.pdf]

CELL JOURNAL (Yakhteh)

Supplementary Information for

Global Scientific Research on SARS-CoV-2 Vaccines: A Bibliometric Analysis

Fakher Rahim, Ph.D.[1], Aida Khakimova, Ph.D.[2*], Ammar Ebrahimi, Ph.D.[3], Oleg Zolotarev, Ph.D.[4], Fatemeh Rafiei Nasab, M.Sc.[5]

1. Thalassemia and Hemoglobinopathy Research Centre, Ahvaz Jundishapur University of Medical Sciences, Ahvaz, Iran
2. Department of Development of Scientific and Innovation Activities, Russian New University, Moscow, Russia
3. Department of Medical Biotechnology, School of Paramedicine, Guilan University of Medical Sciences, Rasht, Iran
4. Department of Information Systems in Economics and Management, Russian New University, Moscow, Russia
5. Department of Scientometrics, Deputy of Research and Technology Affairs, Ahvaz Jundishapur University of Medical Sciences, Ahvaz, Iran

Corresponding Address: Department of Development of Scientific and Innovation Activities, Russian New University, Moscow, Russia
Email: aida_khatif@mail.ru

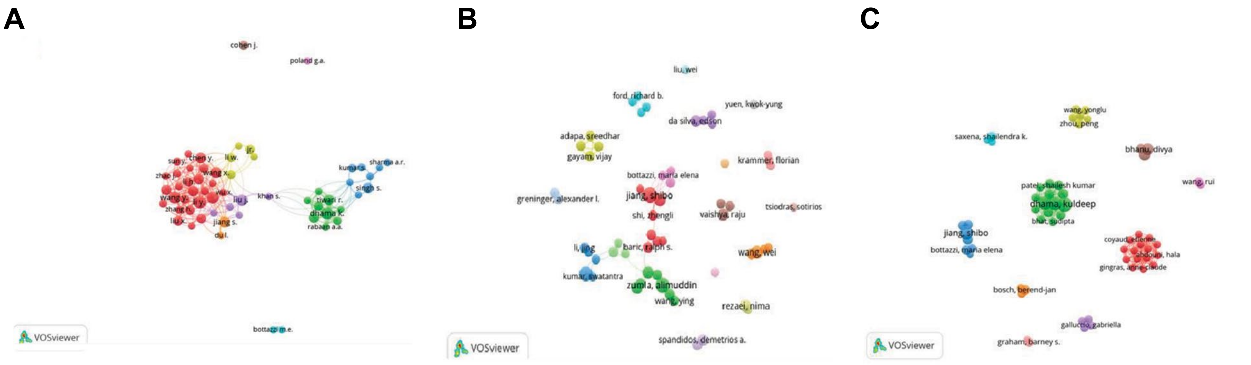

Fig.S1: Co-authorship network for «vaccine+coronavirus» publications 2019-2020. **A.** Scopus database, **B.** PubMed database, and **C.** Dimensions database (by authors).

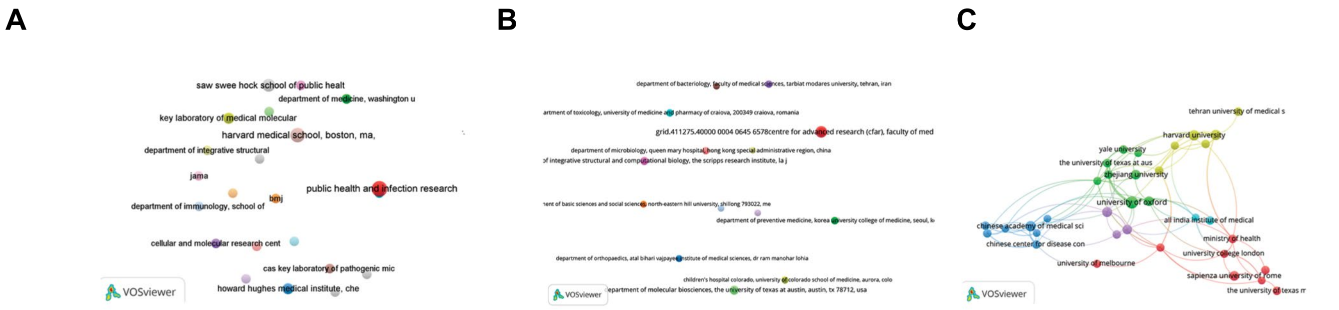

Fig.S2: Co-authorship network for «vaccine+coronavirus» publications 2019-2020. **A.** Scopus database, **B.** PubMed database, and **C.** Dimensions database (by organizations).

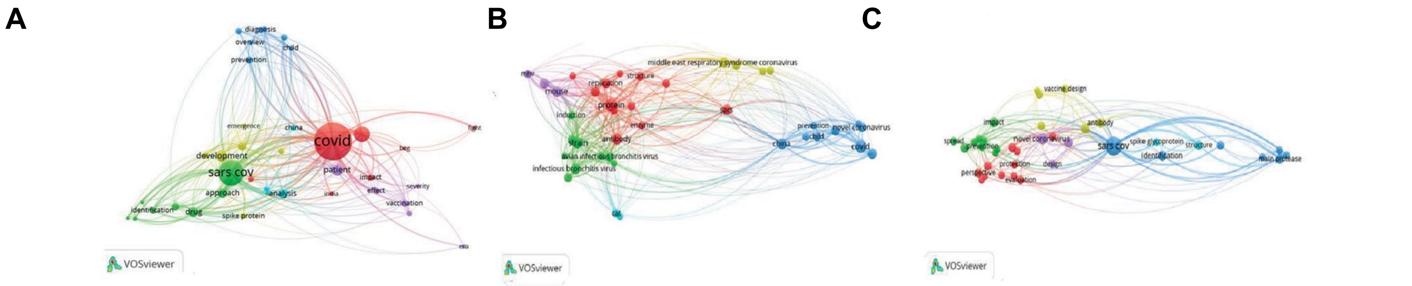

Fig.S3: Co-occurrence network map of 39 keywords for «vaccine+coronavirus» publications 2019-2020. **A.** Scopus database, **B.** PubMed database, and **C.** Dimensions database.

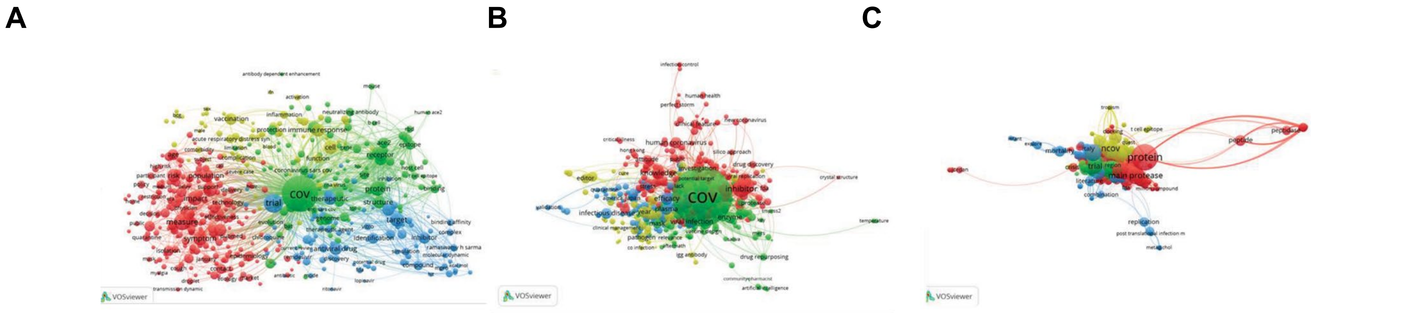

Fig.S4: Co-occurrence network map of 475 keywords for «vaccine+coronavirus» publications 2019-2020. **A.** Scopus database, **B.** PubMed database, and **C.** Dimensions database.

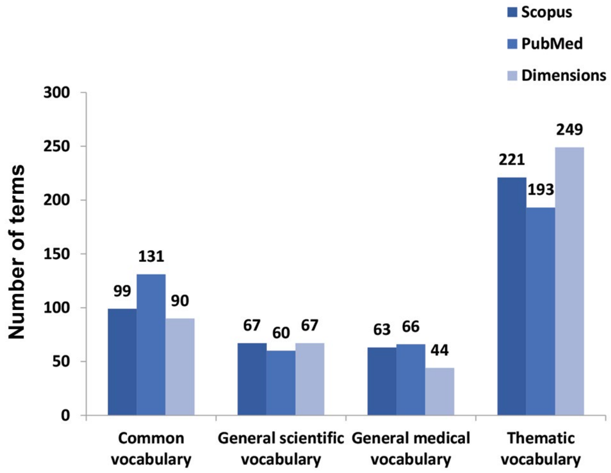

Fig.S5: The relationship of the terms of lexical groups between corpora from different bases.

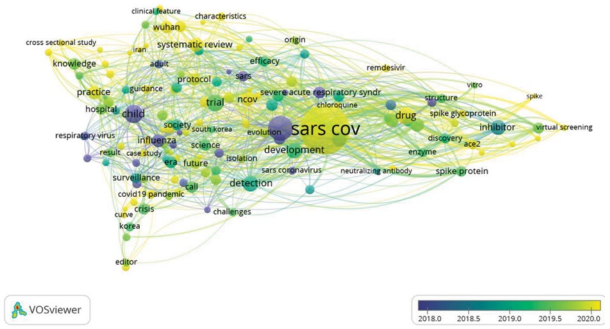

Fig.S6: The most common terms in publications related to research in the field of "vaccine+coronavirus" on the PubMed database, time of appearance.

Table S1: Search strategy for electronic databases

Database	Search strategy
Scopus	TITLE-ABS-KEY("vaccine") OR TITLE-ABS-KEY (vaccines) AND TITLE-ABS-KEY("Wuhan coronavirus") OR TITLE-ABS-KEY("Wuhan seafood market pneumonia virus") OR TITLE-ABS-KEY("COVID19 virus") OR TITLE-ABS-KEY("COVID-19 virus") OR TITLE-ABS-KEY("coronavirus disease 2019 virus") OR TITLE-ABS-KEY("SARS-CoV-2") OR TITLE-ABS-KEY("SARS2") OR TITLE-ABS-KEY("2019-nCoV") OR TITLE-ABS-KEY("2019 novel coronavirus") OR TITLE-ABS-KEY("2019 novel coronavirus infection") OR TITLE-ABS-KEY("COVID19") OR TITLE-ABS-KEY("coronavirus disease 2019") OR TITLE-ABS-KEY("coronavirus disease-19") OR TITLE-ABS-KEY("2019-nCoV disease") OR TITLE-ABS-KEY("2019 novel coronavirus disease") OR TITLE-ABS-KEY("2019-nCoV infection") OR TITLE-ABS-KEY("covid 19") OR TITLE-ABS-KEY ("COVID-19") OR TITLE-ABS-KEY ("COVID-2019") OR TITLE-ABS-KEY ("2019-nCoV") OR TITLE-ABS-KEY ("SARS-CoV-2") OR TITLE-ABS-KEY("2019nCoV")
PubMed	("vaccines"[MeSH Terms] OR "vaccines"[All Fields] OR "vaccine"[All Fields]) AND ("COVID-19"[All Fields] OR "COVID-2019"[All Fields] OR "severe acute respiratory syndrome coronavirus 2"[Supplementary Concept] OR "severe acute respiratory syndrome coronavirus 2"[All Fields] OR "2019-nCoV"[All Fields] OR "SARS-CoV-2"[All Fields] OR "2019nCoV"[All Fields] OR (("Wuhan"[All Fields] AND ("coronavirus"[MeSH Terms] OR "coronavirus"[All Fields])) AND (2019/12[PDAT] OR 2020[PDAT]))) AND ("2019/01/01"[PubDate] : "2020/07/31"[PubDate])
Dimensions	"Vaccine coronavirus" in titles and abstracts

Table S2: Clinical trials on vaccine against COVID-19 disease

No.	Title	Country	Phase	Article type	Vaccine type	Reference No.
1	BCG revaccination of health workers in Brazil to improve innate immune responses against COVID-19: A structured summary of a study protocol for a randomised controlled trial.	Brazil	I/II	Protocol	BCG	RBR-4kjqtg/U1111-1256-3892 (1)
2	Double-Blind, Randomized, Placebo-Controlled Phase III Clinical Trial to Evaluate the Efficacy and Safety of treating Healthcare Professionals with the Adsorbed COVID-19 (Inactivated) Vaccine Manufactured by Sinovac - PROFISCOV: A structured summary of a study protocol for a randomised controlled trial.	Brazil	III	Protocol	Adsorbed vaccine COVID-19 (Sinovac)	NCT0445659 (2)
3	COVID-19 vaccine BNT162b1 elicits human antibody and T_H1 T cell responses		I/II	Full paper	BNT162b1	NCT04380701 (3)
4	Activate: Randomized Clinical Trial of BCG Vaccination against Infection in the Elderly	Greece	III	Full paper	BCG	NCT03296423 (4)
5	Safety and immunogenicity of an rAd26 and rAd5 vector-based heterologous prime-boost COVID-19 vaccine in two formulations: two open, non-randomised phase 1/2 studies from Russia	Russia	I/II	Full paper	rAd26 and rAd5	NCT04436471 and NCT04437875 (5)
6	Effect of an Inactivated Vaccine Against SARS-CoV-2 on Safety and Immunogenicity Outcomes: Interim Analysis of 2 Randomized Clinical Trials	China	I/II	Full paper	SAMC133237/ MN996528	ChiCTR2000031809 (6)
7	Phase I/II study of COVID-19 RNA vaccine BNT162b1 in adults	USA	I/II	Full paper	BNT162b1	NCT04368728 (7)
8	Immunogenicity and safety of a recombinant adenovirus type-5-vectored COVID-19 vaccine in healthy adults aged 18 years or older: a randomised, double-blind, placebo-controlled, phase 2 trial	China	I/II	Full paper	rAd5	NCT04341389 (8)
9	Safety and immunogenicity of the ChAdOx1 nCoV-19 vaccine against SARS-CoV-2: a preliminary report of a phase 1/2, single-blind, randomised controlled trial	UK	I/II	Full paper	ChAdOx1 nCoV-19	NCT04324606 (9)
10	An mRNA Vaccine against SARS-CoV-2 - Preliminary Report	USA	I/II	Full paper	mRNA-1273 SARS-CoV-2	NCT04283461 (10)
11	Safety, tolerability, and immunogenicity of a recombinant adenovirus type-5 vectored COVID-19 vaccine: a dose-escalation, open-label, non-randomised, first-in-human trial.	China	I/II	Full paper	rAd5	NCT04313127 (11)

Table S3: Comparison of co-authorship networks

No.	Index	Scopus	PubMed	Dimensions
1	Total number of authors	6545	28533	10356
2	Selected authors	59	59	59
3	Number of clusters	9	19	10
4	Composition of 1 cluster	Chen J.	Baric, Ralph S.	Abdouni, Hala
		Chen X.	Du, Lanying	Cassonnet, Patricia
		Chen Y.	Jiang, Shibo	Coyaud, Etienne
		Li S.	Li, Wei	Demeret, Caroline
		Liu Y.	Li, Yan	Gingras, Anne-Claude
		Shi Y.	Shaw, Rajib	Jacob, Yves
		Sun J.	Shi, Zhengli	Kim, Dae-Kyum
		Sun Y.	Zhang, Lei	Knapp, Jennifer J.
		Wang J.		Kuang, Da
		Wang Q.		Raught, Brian
		Wang X.		Rayhan, Ashyad
		Wu Y.		Roth, Frederick P.
		Yang Y.		Samavarchi-Tehrani, Payman
		Zhang C.		Sheykhkarimli, Dayag
		Zhang Y.		Van Der Werf, Sylvie
		Zhao J.		
5	Composition of 2 cluster	Chen W.	Bradbury, Jane	Bhat, Sudipta
		Li H.	Heymann, David L	Bonilla-Aldana, D. Katterine
			Ippolito, Giuseppe	
			Perlman, Stanley	
			The Lancet	
			Wang, Ying	
			Zumla, Alimuddin	
		Li J.		Chaicumpa, Wanpen
		Li X.		Dhama, Kuldeep
		Li Y.		Malik, Yashpal Singh
		Liu J.		Patel, Shailesh Kumar
		Liu X.		Pathak, Mamta
		Wang H.		Rabaan, Ali A.
		Wang L.		Rodriguez-Morales, Alfonso J.
		Wang Y.		Sah, Ranjit
		Wu X.		Sharun, Khan
		Yang X.		Singh, Karam Pal
		Zhang H.		Sircar, Shubhankar
		Zhang S.		Tiwari, Ruchi
6	Composition of 3 cluster	Bonilla-Aldana D.K.	Kumar, Swatantra	Bottazzi, Maria Elena
		Dhama K.	Li, Jing	Du, Lanying
		Khan S.	Saxena, Shailendra K.	Hotez, Peter J.
		Malik Y.S.	Wang, Quanyi	Jiang, Shibo
		Patel S.K.	Zhang, Yi	Lu, Lu
		Rabaan A.A.		Strych, Ulrich
		Rodriguez-Morales A.J.		Xia, Shuai
		Sah R.		
		Sharun K.		
		Tiwari R.		

Table S3: Continued

No.	Index	Scopus	PubMed	Dimensions
7	Composition of 4 cluster	Gupta N.<br>Kumar P.<br>Kumar S.<br>Sharma A.R.<br>Singh A.<br>Singh R.<br>Singh S.<br>Singh S.K.	Adapa, Sreedhar<br>Gayam, Vijay<br>Konala, Venu Madhav<br>Naramala, Srikanth	Liu, Xinsheng<br>Wang, Yonglu<br>Zhang, Liping<br>Zhang, Yongguang<br>Zhao, Donghong<br>Zhou, Peng
8	Composition of 5 cluster	Haagmans B.L.<br>Jr. Li W.<br>Li Z.<br>Perlman S.	Bonilla-Aldana, D. Katterine<br>Da Silva, Edson<br>Rodriguez-Morales, Alfonso J.<br>Sah, Ranjit	Galluccio, Gabriella<br>Magazzino, Cosimo<br>Putrino, Alessandra<br>Raso, Mario
9	Composition of 6 cluster	Bottazzi M.E.<br>Hotez P.J.	Ford, Richard B.<br>Mazzaferro, Elisa M.<br>Strauss, Ellen G.<br>Strauss, James H.	Kumar, Swatantra<br>Maurya, Vimal K.<br>Saxena, Shailendra K.
10	Composition of 7 cluster	Du L.<br>Jiang S.	Chen, Wei<br>Liu, Yang<br>Wang, Wei<br>Zhang, Jun	Bosch, Berend-Jan<br>Haagmans, Bart L.<br>Li, Wentao
11	Composition of 8 cluster	Cohen J.	Haleem, Abid<br>Javaid, Mohd<br>Vaishya, Raju	Alluri, Anjani<br>Bhanu, Divya<br>Shanker, Arun
12	Composition of 9 cluster	Poland G.A.	Bottazzi, Maria Elena<br>Hotez, Peter J.<br>Strych, Ulrich	Wang, Rui<br>Wei, Guo-Wei
13	Composition of 10 cluster	-	Krammer, Florian<br>Poland, Gregory A.<br>Veesler, David	Graham, Barney S.<br>Zhang, Yi
14	Composition of 11 cluster	-	Graham, Barney S.<br>Mclellan, Jason S.<br>Wrapp, Daniel	-
15	Composition of 12 cluster	-	Greninger, Alexander L.<br>Jerome, Keith R.	-
16	Composition of 13 cluster	-	Rezaei, Nima<br>Sahu, Kamal Kant	-
17	Composition of 14 cluster	-	Spandidos, Demetrios A.<br>Tsatsakis, Aristidis	-
18	Compositions of other clusters		Liu, Wei<br>Shoenfeld, Yehuda<br>Tsiodras, Sotirios<br>Wang, Xinghuan<br>Yuen, Kwok-Yung	-

Table S4 : Data on co-authorship networks by organiztations obtained by corpora from Scopus ,PubMed ,and Dimensions

No.	Index	Scopus	PubMed	Dimensions
1	Total number of organizations	5185	15718	1684
2	Selected organizations	34 (at least 3 publications)	34 (at least 3 publications)	34 (at least 12 publications)
3	Number of clusters	22	14	6
4	Composition of 1 cluster	Tribhuvan University Teaching Hospital, Institute of Medicine, Kathmandu, Nepal<br>Icar-Indian Veterinary Research Institute, Izatnagar, Bareilly, Uttar Pradesh, India (division of pathology, division of surgery)<br>Fundacion Universitaria Autonoma de Las Americas, Pereira, Risaralda, Colombia (Faculty of Medicine, Faculty of Health Sciences, Semillero de Zoonosis)<br>Johns Hopkins Aramco healthcare, Dhahran, Saudi Arabia-	Barts and The London School of Medicine and Dentistry, Queen Mary University of London, United Kingdom<br>Barts Health Nh's Trust, London, United Kingdom<br>King George's Medical University (Kgmu), Lucknow, India (Centre for Advanced Research (Cfar), Cfar-Stem Cell/Cell Culture Unit)<br>Ucl Medical School, University College London, United Kingdom	Icahn School of Medicine At Mount Sinai<br>King Abdul-Aziz University<br>King Saud University<br>Ministry of Health<br>Sapienza University of Rome<br>The University of Texas Medical Branch at Galveston<br>University College London<br>University of Melbourne
5	Composition of 2 cluster	Washington University School of Medicine, st. Louis, United States (Department of Medicine, Department of Molecular Microbiology, Department of Pathology & Immunology)	Korea University College of Medicine, Seoul, Korea<br>Children's Hospital of Chongqing Medical University, Chongqing 400014, China<br>Lanzhou University, Lanzhou, China (School of Basic Medical Sciences, The First School of Clinical Medicine)<br>Korea Institute of Oriental Medicine, Daejeon, Korea	National Institute of Allergy and Infectious Diseases<br>The Ohio State University<br>The University of Texas at Austin<br>University of Hong Kong<br>University of North Carolina At Chapel Hill<br>University of Oxford<br>Yale University<br>Zhejiang University
6	Composition of 3 cluster	Yale University School of Medicine, New Haven, Ct, United States<br>Howard Hughes Medical Institute, Chevy Chase, Md, United States	Atal Bihari Vajpayee Institute of Medical Sciences, Dr Ram Manohar Lohia Hospital, New Delhi, India<br>Indraprastha Apollo Hospital, Saritavihar, Mathura Road, New Delhi, India<br>Southport and Ormskirk Nh's Trust, Southport, Pr8 6Pn, United Kingdom	Chinese Academy of Medical Sciences & Peking Union Medical College<br>Chinese Center for Disease Control and Prevention<br>Huazhong University Of Science and Technology<br>Tsinghua University<br>University of Chinese Academy of Sciences<br>University of Minnesota<br>Wuhan Institute of Virology
7	Composition of 4 cluster	School of Basic Medical Sciences, Fudan University, Shanghai, China-<br>Lindsey f. Kimball Research Institute, New York Blood Center, New York, Ny, United States	University of Colorado School of Medicine, Aurora, Colo<br>The Ohio State University School of Medicine, Columbus, Ohio<br>Umdmj Rutgers University School of Medicine, Newark, Nj	Harvard University<br>Johns Hopkins University<br>Tehran University of Medical Sciences<br>University of Michigan<br>University of Pennsylvania<br>University of Washington
8	Composition of 5 cluster	Shahrekord University of Medical Sciences, Shahrekord, Iran<br>Baqiyatallah University of Medical Sciences, Tehran, Iran	Tarbiat Modares University, Tehran, Iran<br>The Australian National University, Canberra, Australia	Fudan University<br>National University of Singapore<br>Shanghai Jiao Tong University
9	Composition of 6 cluster	Mahidol University, Bangkok, Thailand<br>Razi Vaccine and Serum Research Institute, Agricultural Research, Education and Extension Organization (Areeo), Karaj, Iran	University of Medicine and Pharmacy of Craiova, Craiova, Romania (Department of Clinical Pharmacy, Department of Toxicology)	all India Institute of Medical Sciences<br>Imperial College London

Table S5: Keywords co-occurring in «vaccine + coronavirus» publications 2019-2020 on the Scopus database (rank based on total link strength)

No. of cluster (items)	Top 10 keywords	Links	Total link strength	Occurrences
1 (8)	Covid	38	682	886
	pandemic	27	194	162
	impact	14	39	26
	insight	16	33	22
	case	13	25	16
	india	8	23	13
	bcg	8	17	10
	fight	4	16	13
2 (8)	sars cov	36	339	395
	approach	22	87	55
	drug	17	75	56
	target	15	60	36
	identification	15	55	27
	main protease	9	35	15
	potential inhibitor	8	22	10
	molecular docking	10	19	10
3 (7)	management	12	52	25
	diagnosis	12	49	28
	prevention	15	49	33
	pathogenesis	11	43	28
	epidemiology	12	34	18
	child	12	30	17
	overview	12	27	17
4 (6)	development	17	99	82
	novel coronavirus	26	75	47
	ncov	13	31	26
	spike protein	10	30	21
	severe acute respiratory syndrome coronavirus	10	21	22
	emergence	8	18	12
5 (6)	patient	25	130	79
	vaccination	12	46	33
	effect	13	40	27
	mortality	9	37	20
	severity	10	23	13
	era	5	14	12
6 (4)	analysis	23	95	59
	systematic review	13	36	18
	efficacy	10	27	16
	china	14	23	21

Table S6: Keywords co-occurring in «vaccine + coronavirus» publications 2019-2020 on the Dimensions database (rank based on total link strength)

No of cluster (items)	Top 10 keywords	Links	Total link strength	Occurrences
1 (11)	evaluation	9	36	28
	challenge	13	34	33
	characterization	13	31	31
	china	9	19	40
	efficacy	9	18	23
	perspective	10	18	36
	protection	5	18	26
	porcine epidemic diarrhea virus	7	15	20
	infectious bronchitis virus	4	11	25
	mer	6	8	15
	coronavirus vaccine	4	4	25
2 (9)	covid19	7	10	22
	diagnosis	12	41	32
	epidemiology	12	32	27
	impact	11	18	43
	management	12	40	43
	overview	8	14	23
	prevention	14	50	46
	sarscov2	8	12	22
	spread	5	13	29
3 (8)	Sars Cov	33	370	631
	Main Protease	9	83	45
	Identification	15	67	54
	Virtual Screening	7	45	24
	Molecular Docking	11	42	20
	Potential Inhibitor	9	35	17
	Drug Repurposing	7	34	22
	Prediction	8	30	28
4 (6)	spike protein	18	69	55
	antibody	7	49	45
	severe acute respiratory syndrome coronavirus	14	30	32
	middle east respiratory syndrome coronavirus	9	20	26
	vaccine design	5	19	27
	mers cov	7	18	21
5 (3)	novel coronavirus	23	107	86
	ncov	15	61	52
	design	13	36	29
6 (2)	structure	11	52	45
	spike glycoprotein	7	31	26

Table S7: Keywords co-occurring in «vaccine + coronavirus» publications 2019-2020 on the PubMed database (rank based on total link strength)

No of cluster (items)	Top 10 keywords	Links	Total link strength	Occurrences
1 (12)	protein	36	633	1170
	antibody	34	378	505
	expression	28	269	376
	replication	29	228	403
	monoclonal antibody	27	160	194
	structure	23	160	259
	sars	22	148	422
	nucleocapsid protein	23	144	239
	enzyme	24	133	163
	inhibition	19	113	140
	protease	19	85	209
	murine coronavirus	14	56	134
2 (9)	strain	29	476	636
	infectious bronchitis virus	17	327	548
	chicken	14	188	233
	transmissible gastroenteritis virus	21	156	291
	protection	30	140	156
	induction	22	136	145
	avian infectious bronchitis virus	18	121	229
	pathogenicity	23	108	107
	swine	12	65	110

Table S7: Continued

No of cluster (items)	Top 10 keywords	Links	Total link strength	Occurrences
3 (9)	china	26	416	535
	covid	20	405	980
	coronavirus disease	14	267	425
	novel coronavirus	15	216	329
	pneumonia	20	197	300
	ncov	15	127	136
	child	15	111	273
	prevention	18	74	122
	respiratory virus	14	57	162
4 (4)	middle east respiratory syndrome coronavirus	20	224	439
	mers cov	22	214	313
	mer	16	107	165
	middle east respiratory syndrome	14	81	206
5 (3)	mouse	25	401	556
	mouse hepatitis virus	18	375	477
	mhv	17	207	204
6 (2)	cat	15	98	202
	feline infectious peritonitis	14	75	139

Table S8: Keywords co-occurring in «vaccine + coronavirus» publications 2019-2020 on the Scopus database (rank based on total link strength)

No of cluster (items)	Top 20 keywords	Links	Total link strength	Occurrences
1 (212)	Measure	415	2184	180
	Symptom	411	1887	132
	Population	391	1583	125
	Health	399	1474	132
	Impact	376	1412	136
	Risk	359	1341	127
	Community	371	1195	110
	Intervention	367	1188	94
	Age	338	1149	84
	Fever	334	1042	63
	Contact	342	1032	69
	Social Distancing	303	987	72
	Isolation	312	930	67
	Epidemiology	317	875	69
	Care	313	874	74
	Practice	304	799	68
	Status	319	782	65
	Period	270	697	56
	Government	277	647	60
	Technology	261	631	63
2 (107)	Cov	475	9539	859
	Protein	395	2063	137
	Receptor	374	1856	118
	Enzyme	346	1496	93
	Structure	346	1411	100
	Antibody	361	1383	100
	Ace2	312	1304	82
	Mers Cov	369	1280	88
	Spike Protein	325	1263	85
	Region	355	1197	85
	Receptor Binding Domain	256	949	53
	Origin	322	937	69
	Site	308	929	59
	Binding	247	908	54
	Sequence	302	906	63
	Epitope	225	895	67
	Entry	274	845	50
	Genome	291	816	56
	Spike	230	816	56
	Mer	301	789	63
3 (90)	Trial	464	2769	245
	Target	371	1828	129

Table S8: Continued

No of cluster (items)	Top 20 keywords	Links	Total link strength	Occurrences
	Interaction	372	1608	102
	Therapeutic	378	1393	109
	Inhibitor	302	1371	87
	Antiviral Drug	365	1341	106
	Identification	337	1168	83
	Compound	257	994	64
	Database	311	832	57
	Molecule	265	817	59
	Candidate	278	751	57
	Investigation	307	730	53
	Causative agent	296	721	49
	Remdesivir	279	706	48
	Simulation	224	697	43
	Screening	273	635	43
	Ramaswamy H Sarma	174	608	32
	Protease	234	590	32
	Acid	267	586	41
	Main Protease	154	573	32
4 (66)	Cell	396	1707	125
	Immune response	368	1483	118
	Vaccination	329	965	95
	Function	310	793	58
	Acute respiratory distress syndrome	268	708	47
	Lung	261	665	47
	Cytokine storm	252	635	46
	Immune system	256	584	44
	Inflammation	237	569	42
	Complication	249	538	43
	T Cell	214	530	38
	Severe Covid	243	489	37
	Immunization	211	438	40
	Comorbidity	216	432	29
	Expression	200	419	28
	Rna Virus	219	409	28
	Activation	194	408	27
	Risk Factor	206	392	25
	Cytokine	193	371	26
	Ards	196	351	22

Table S9: Keywords co-occurring in «vaccine + coronavirus» publications 2019-2020 on the PubMed database (rank based on total link strength)

Number of cluster (items)	Top 20 keywords	Links	Total link strength	Occurrences
1 (191)	inhibitor	47	116	45
	main protease	34	93	28
	identification	34	84	39
	knowledge	27	64	24
	structure	23	58	18
	protein	38	54	29
	characterization	37	53	22
	spike glycoprotein	20	46	17
	discovery	22	32	13
	virtual screening	19	32	10
	attitude	12	31	12
	investigation	21	31	15
	molecular docking	16	30	8
	cross sectional study	14	29	10
	potential inhibitor	14	27	8
	spike	10	24	5
	porcine deltacoronavirus	19	23	10
	infectivity	15	22	10
	expression	14	21	10
	retrospective study	17	21	9
2 (104)	cov	246	756	510
	spike protein	28	69	24
	design	21	40	18
	receptor	26	39	19
	enzyme	24	38	20
	epitope	17	36	17
	receptor binding domain	17	27	6
	entry	15	26	9
	protease	19	25	10
	remdesivir	19	25	15
	binding	15	22	8
	neutralization	9	18	3
	saudi arabia	18	18	6
	human monoclonal antibody	11	16	3
	middle east respiratory syndrome coronavirus	14	16	9
	monoclonal antibody	13	16	7
	drug repurposing	10	15	8
	production	10	15	10
	mask	9	14	12

Table S9: Continued

Number of cluster (items)	Top 20 keywords	Links	Total link strength	Occurrences
	mers cov	8	14	7
3 (91)	target	31	51	25
	inhibition	26	40	14
	efficacy	25	37	23
	neutralizing antibody	13	30	11
	plasma	16	29	17
	virus infection	22	26	21
	viral infection	21	25	21
	stress	14	16	8
	acute respiratory distress syndrome	9	14	10
	immunotherapy	10	13	13
	infectious disease	13	13	17
	study protocol	9	13	6
	animal	11	12	6
	anxiety	7	12	4
	vulnerability	11	12	6
	immunogenicity	9	11	8
	quality	9	11	8
	randomised controlled trial	7	11	5
	reduction	8	11	8
	animal model	7	10	3
4 (76)	respiratory virus	20	25	16
	vitro	16	25	9
	editor	11	23	14
	letter	11	23	14
	replication	18	22	13
	health care worker	16	21	14
	human	14	21	9
	year	19	21	20
	incidence	15	18	10
	introduction	15	15	7
	mouse	10	15	9
	igg antibody	8	14	5
	interferon	10	14	6
	etiology	11	13	10
	pathogen	10	13	12
	pcr	9	13	11
	sars coronavirus	13	13	13
	acute respiratory infection	8	11	10
	april	11	11	4
	hospitalized child	10	11	8

Table S10: Keywords co-occurring in «vaccine + coronavirus» publications 2019-2020 on the Dimensions database (rank based on total link strength)

Number of cluster (items)	Top 20 keywords	Links	Total link strength	Occurrences
1 (187)	protein	95	231	103
	main protease	42	120	45
	peptide	24	97	17
	peptidase	12	83	13
	homology modelling	10	80	11
	noncovalent lead inhibitor	10	80	11
	possible antiviral properties	10	80	11
	structure	44	80	41
	virtual screening	28	77	24
	molecular docking	28	74	20
	efficacy	37	62	23
	potential inhibitor	28	60	17
	inhibitor	39	58	29
	protein ligand interaction	7	48	7
	spike glycoprotein	27	43	26
	whole genome sequence analysis	7	42	6
	discovery	17	36	13
	drug repurposing	17	35	19
	comparison	19	32	12
	novel coronavirus covid	8	32	8
2 (95)	characterization	39	55	31
	strain	33	54	25
	trial	38	54	38
	safety	31	53	16
	systematic review	31	52	25
	immunogenicity	25	43	13
	porcine epidemic diarrhea virus	26	43	20
	infectious bronchitis virus	22	38	21
	middle east respiratory syndrome coronavirus	24	37	26
	mer	20	32	15
	sars	18	31	14
	gene	28	30	16
	mers cov	19	30	21
	binding	18	29	12
	vaccine candidate	20	28	16
	protection	17	26	26
	pathogenicity	16	25	12
	open label	11	23	5
	phase	12	17	7

Table S10: Continued

Number of cluster (items)	Top 20 keywords	Links	Total link strength	Occurrences
	piglet	12	17	7
3 (93)	management	25	68	43
	knowledge	25	66	19
	evaluation	37	64	28
	spread	21	52	29
	italy	14	46	14
	clinical experience	9	39	7
	literature	17	38	16
	contagion	6	36	7
	italian dentist	6	36	6
	mortality	18	32	17
	combination	13	25	9
	morbidity	10	25	11
	vitro	11	25	12
	enzyme	20	24	17
	natural compound	13	22	7
	replication	13	22	14
	block	6	18	3
	chiral anti cov drugs	6	18	3
	computational	6	18	3
	eliminate sars cov	6	18	3
	assessment	15	16	11
4 (84)	ncov	53	92	52
	use	31	60	23
	vaccine design	32	58	27
	epitope	33	55	30
	prediction	30	54	25
	diagnosis	27	43	32
	design	26	42	26
	chicken	14	32	12
	silico approach	20	25	10
	informational spectrum methodology	8	24	3
	natural reservoir	8	24	3
	novel coronavirus disease	17	24	14
	potential receptor	8	24	3
	rapid biological analysis	8	24	3
	therapeutic vaccine target	8	24	3
	tropism	8	24	4
	glycoprotein	12	23	8
	peptide vaccine	12	23	10
	computational approach	11	22	7
	immunoinformatic	10	20	7

Table S11: Comparison of coronavirus diseases names co-occurring in «vaccine+coronavirus» publications 2019-2020 on the Scopus, PubMed, Dimensions databases

Terms	Scopus	PubMed	Dimensions
Main terms common to all corpora	Acute respiratory distress syndrome, corona virus, corona virus disease, coronavirus sars cov, mer, mers, mers cov, middle east respiratory syndrome, middle east respiratory syndrome coronavirus, novel, novel coronavirus pneumonia, sar, sars coronavirus		
Main terms common to Scopus and PubMed	COV		
terms common to PubMed and Dimensions		New coronavirus, novel coronavirus covid, novel coronavirus SARS CoV, porcine deltacoronavirus, porcine epidemic diarrhea virus, SARS CoV2, SARSCoV2 infection, severe acute respiratory syndrome	
Main specific terms from each corpus	Betacoronavirus, contagious disease, HCoV, MERS CoV infection, NCoV infection, novel corona virus, novel SARS CoV, novel virus, pathogenic coronavirus	Avian coronavirus, avian infectious bronchitis virus, china coronavirus, coronavirus covid, covid19, IBV, infectious bronchitis virus, ncov, novel coronavirus disease, pandemic coronavirus disease, pandemic covid, pandemic novel coronavirus, PEDV, SARS, SARS CoV2 SARS CoV2 infection	Acute respiratory infection, corona, footandmouth disease virus, human coronavirus, human metapneumovirus, influenzalike illness, MERSCoV, novel coronavirus infection, novel covid, porcine epidemic diarrhoea virus, respiratory viral infection, respiratory virus, respiratory virus infection, severe acute respiratory infection, severe coronavirus disease, viral respiratory infection

Table S12 : Comparison of specific terms related to vaccine co-occurring in» vaccine+coronavirus» publications 2019-2020 on the Scopus, PubMed, Dimensions databases

**Terms**	**Scopus**	**Dimensions**	**PubMed**
Main terms common to all corpora	Animal model, anti sars cov, antiviral agent, binding, coronavirus vaccine, crystal structure, drug discovery, drug repurposing, enzyme, epitope, genome, immune system, immunogenicity, immunoinformatics approach, inhibition, inhibitor, interferon, main protease, molecular docking, molecular dynamic, molecular dynamics simulation, mouse, mpro, neutralizing antibody, nucleocapsid protein, pathogenicity, peptide, protease, protective immunity, protein, receptor, receptor binding, receptor binding domain, remdesivir, replication, repurposing, s protein, spike, spike glycoprotein, tmprss2, transmission dynamic, vaccine design, vaccines, virtual screening		
Main specific terms from each corpus	ACE2 (receptor), active site, angiotensin, antibiotic, antibody, antibody dependent enhancement, b cell, bind, binding affinity, candidate vaccine, CD4, cell, chloroquine, convalescent plasma, corticosteroid, drug administration, effective drug, epidemiology, favipiravir, genome sequence, host cell, human ace2, immune response, incubation period, infection rate, inflammation, inflammatory cytokine, inflammatory influenza, intermediate host, lopinavir, lopinavir ritonavir, lymphopenia, new drug, new vaccine, pathology, repurposed drug, ritonavir, RNA virus, screening, sequence, severe case, severe covid, severe disease, severe illness, specific antiviral treatment, vaccination, vaccine trial, viral entry, viral genome, virulence, virus replication	3clpro protein target, ace2 receptor, animal models, antibody testing, antigenicity, aptamers targeting receptor binding domain, b cell epitopes, bacillus calmette gurin, BCG vaccination (policy), chicken, chiral anti cov drugs, codon usage, combat covid, conformational change, coronavirus spike, coronavirus vaccine development, deep docking, dromedary camel, e pharmacophore, e protein, effective vaccine, molecular orbital calculation, genomic diversity, genomic landscape, glycan, homology modeling, human ace2 receptor, ifn, immune enhancement, immunoinformatic(s) (study), inactivated vaccine candidate, infected cell, influenza vaccine, informational spectrum methodology, inhibitors, intermolecular interaction, main protease enzyme, metadichol, mitigation strategy, molecular docking approach, monkey, msuppro sup, multi epitope (vaccine), n terminal domain, ncov vaccine, hydroxyethylamine analog, mutation, noncovalent lead inhibitor, novel coronavirus protease identified, novel coronavirus spike protein, nucleocapsid, peptidase, peptide vaccine, piglet, possible antiviral properties, post translational infection mechanism, potent inhibitor, potential antiviral drug, potential peptide inhibitor, potential receptor, spike, protease inhibitor(s) designed, protein ligand interaction quantum mechanical scoring, rdrp, reverse vaccinology approach, rna dependent rna polymerase, sars cov2 enzyme inhibitors targeting viral attachment, protein ligand interaction, silico analysis (discovery), silico drug repurposing, silico identification, silico screening, subunit vaccine, targeting sars cov, therapeutic vaccine target, treat covid, vaccine target, virtual screening driven drug discovery, whole genome sequence (analysis)	Acute respiratory infection, antiviral therapy, biosensor, co infection, covid19 outbreak, DNA vaccine, dog, drug delivery, ECDC, epidemiological, extracellular vesicle, gene therapy, global emergency, igg antibody, igm, immune checkpoint inhibitor, immunology, immunopathology, infectious diseases, infection control, infection prevention, influenza vaccination, infodemic, ivermectin, macrophage, mesenchymal stem cell, microbe, molecular characterization, novel coronavirus outbreak, nucleic acid, predictor, receptor ace2,<br><br>rna dependent rna polymerase, ruxolitinib, serology, seroprevalence, serum, statin, viral, viral etiology, viral rna

References

1. Junqueira-Kipnis AP, Dos Anjos LRB, Barbosa LCS, da Costa AC, Borges KCM, Cardoso A, et al. BCG revaccination of health workers in Brazil to improve innate immune responses against COVID-19: a structured summary of a study protocol for a randomised controlled trial. Trials. 2020; 21(1): 881.
2. Palacios R, Patiño EG, de Oliveira Piorelli R, Conde MTRP, Batista AP, Zeng G, et al. Double-blind, randomized, placebo-controlled phase III Clinical trial to evaluate the efficacy and safety of treating healthcare professionals with the adsorbed COVID-19 (Inactivated) vaccine manufactured by Sinovac - PROFISCOV: a structured summary of a study protocol for a randomised controlled trial. Trials. 2020; 21(1): 853.
3. Sahin U, Muik A, Derhovanessian E, Vogler I, Kranz LM, Vormehr M, et al. COVID-19 vaccine BNT162b1 elicits human antibody and TH1 T cell responses. Nature. 2020; 586(7830): 594-599.
4. Giamarellos-Bourboulis EJ, Tsilika M, Moorlag S, Antonakos N, Kotsaki A, Domínguez-Andrés J, et al. Activate: randomized clinical trial of BCG vaccination against infection in the elderly. Cell. 2020; 183(2): 315-323.e9.
5. Logunov DY, Dolzhikova IV, Zubkova OV, Tukhvatullin AI, Shcheblyakov DV, Dzharullaeva AS, et al. Safety and immunogenicity of an rAd26 and rAd5 vector-based heterologous prime-boost COVID-19 vaccine in two formulations: two open, non-randomised phase 1/2 studies from Russia. Lancet. 2020; 396(10255): 887-897.
6. Xia S, Duan K, Zhang Y, Zhao D, Zhang H, Xie Z, et al. Effect of an inactivated vaccine against SARS-CoV-2 on safety and immunogenicity outcomes: interim analysis of 2 randomized clinical trials. JAMA. 2020; 324(10): 951-960.
7. Mulligan MJ, Lyke KE, Kitchin N, Absalon J, Gurtman A, Lockhart S, et al. Phase I/II study of COVID-19 RNA vaccine BNT162b1 in adults. Nature. 2020; 586(7830): 589-593.
8. Zhu FC, Guan XH, Li YH, Huang JY, Jiang T, Hou LH, et al. Immunogenicity and safety of a recombinant adenovirus type-5-vectored COVID-19 vaccine in healthy adults aged 18 years or older: a randomised, double-blind, placebo-controlled, phase 2 trial. Lancet. 2020; 396(10249): 479-488.
9. Folegatti PM, Ewer KJ, Aley PK, Angus B, Becker S, Belij-Rammerstorfer S, et al. Safety and immunogenicity of the ChAdOx1 nCoV-19 vaccine against SARS-CoV-2: a preliminary report of a phase 1/2, single-blind, randomised controlled trial. Lancet. 2020; 396(10249): 467-478.
10. Jackson LA, Anderson EJ, Rouphael NG, Roberts PC, Makhene M, Coler RN, et al. An mRNA Vaccine against SARS-CoV-2 - preliminary report. N Engl J Med. 2020; 383(20): 1920-1931.
11. Zhu FC, Li YH, Guan XH, Hou LH, Wang WJ, Li JX, et al. Safety, tolerability, and immunogenicity of a recombinant adenovirus type-5 vectored COVID-19 vaccine: a dose-escalation, open-label, non-randomised, first-in-human trial. Lancet. 2020; 395(10240): 1845-1854.
